# Topography of the Anatomical Landmarks of Carotid Bifurcation and Clinical Significance

**DOI:** 10.7759/cureus.31715

**Published:** 2022-11-20

**Authors:** Ömer Faruk Ci̇han, Kübra Deveci̇

**Affiliations:** 1 Anatomy, Gaziantep University, Gaziantep, TUR

**Keywords:** thyroid cartilage, hyoid bone, gonion, mastoid process, cervical vertebrae, carotid bifurcation

## Abstract

Objective: Carotid bifurcation (CB) and its terminal branches are the most common sites of atherosclerotic plaques. In surgical treatment, these plaques can be reached by an endarterectomy technique. The success of the technique can be achieved with good anatomical knowledge of these arteries and their relationships with surrounding structures.

Materials and methods: The study was performed retrospectively on archived images of patients with computed tomography angiography (CTA). Two hundred forty-seven patients who met the criteria were included in this study. Three-dimensional (3D) reconstructions of two-dimensional CTA images were made automatically using the open-source software Horos v.4.0.0. The distance between the transverse plane passing through the bifurcation point (BP) and the defined planes of the surrounding structures was evaluated.

Results: CB was observed below the mastoid process, gonion point, and hyoid bone. CB was observed above the thyroid cartilage. Carotid bifurcation was seen at 15 levels in total, the lowest in the upper 1/3 of the C6 vertebral body and the highest in the lower 1/3 of the C2 vertebral body. In all cases, the most common level was the C3 lower level.

Conclusion: All these values, which emerged as a result of the study, provide general information about the topography of the CB according to the neighboring structures. Estimating the location of the CB according to the gonion and hyoid bone will give a more accurate result. The vertebral level on the right side increased in direct proportion to age; there was no similar relationship on the left side. It is necessary to be aware of these anatomical variations in order to prevent various iatrogenic complications.

## Introduction

The common carotid artery (CCA) forms the carotid bifurcation (CB) in the neck region, divides into its terminal branches, the internal carotid artery (ICA), and the external carotid artery (ECA), and provides head and neck arterial nutrition. It is an important criterion to externally determine the CB level during the clinical examination of the neck [1].

Accurate determination of the CB level can also be useful in planning surgical interventions and estimating technical difficulties. Carotid endarterectomy is very dangerous and requires meticulous dissection in a complex region [2,3]. Especially high levels of CBs are an important factor that complicates neck dissection and carotid endarterectomies [4]. In addition, studies have warned surgeons about the vulnerable hypoglossal nerve and superior thyroid artery, which may originate from the CCA in the presence of high levels of CB [5-7]. In low-level CBs, the course of two arteries instead of one on the ipsilateral side of the neck will complicate the surgical intervention to be applied to this region [8].

The carotid arteries, which are the primary vessels feeding, and the jugular veins, which provide venous drainage to the head and neck, are located in the anterolateral neck, but these important structures do not have protective bone structures. For this reason, it is the large structures that are most injured in penetrating injuries of the neck [9,10].

Carotid bifurcation and its terminal branches are the most common sites of atherosclerotic plaques [11]. Cerebrovascular diseases are one of the most common causes of death in developed countries, and in 15-20% of these cases, atherosclerotic lesions in the CB are to blame [12]. Atherosclerosis is characterized by increased thickness of the tunica intima-media in the entire arterial network. Measurement of thickness over the carotid arteries with the aid of noninvasive procedures can give an idea of the degree of atherosclerosis in the entire arterial network. In surgical treatment, these plaques can be reached by an endarterectomy technique. The success of the technique can be achieved with good anatomical knowledge of these arteries and their relationships with surrounding structures [13-15].

Superficial anatomical signs are very important for clinical examination, radiological imaging, diagnosis, treatment planning, surgical interventions, and understanding of anatomy [16,17]. The locations of the deep structures in the neck region can be described according to the anatomical formations that can be easily observed from the outside. The most frequently used ones are the hyoid bone, thyroid cartilage, and cervical vertebrae, but the less frequently used structures include the gonion and mastoid processes [16]. This study aimed to examine the topographical relationship of the CB with the surrounding anatomical structures. The changes in the obtained data according to age, gender, and right-left side were evaluated. We believe that it can provide useful information to operators who will attempt surgery in this region.

## Materials and methods

Study population

The study was carried out retrospectively on archived images of patients with head-neck and brain-carotid CTA for various reasons at Gaziantep University Medical Faculty Hospital between 2015 and 2020. For our purposes, 400 images from approximately 4000 patients obtained through archive scanning were analyzed. As a result, a total of 247 patients who met the criteria were included in this study (Table 1).

**Table 1:** Distribution of the age of the individuals included in the study by gender.

Gender	Age		
	n	%	Mean±SD (min–max)
Female	123	49.8	51.75±17.8 (19–91)
Male	124	50.2	55.96±17.02 (18–95)
Total	247	100	53.87±17.51 (18–95)

Acquisition and Processing of Images

Three-dimensional reconstructions of two-dimensional CTA images were made automatically using the open source software Horos v.4.0.0 and the relevant measurements were made ready to be made.

Ethics Statement

The study was conducted in accordance with the principles of the Declaration of Helsinki, and prior permission was obtained from the Clinical Research Ethics Committee of the University of Gaziantep (decision no: 2019/289).

Measurements from the lateral side

**Figure 1:**
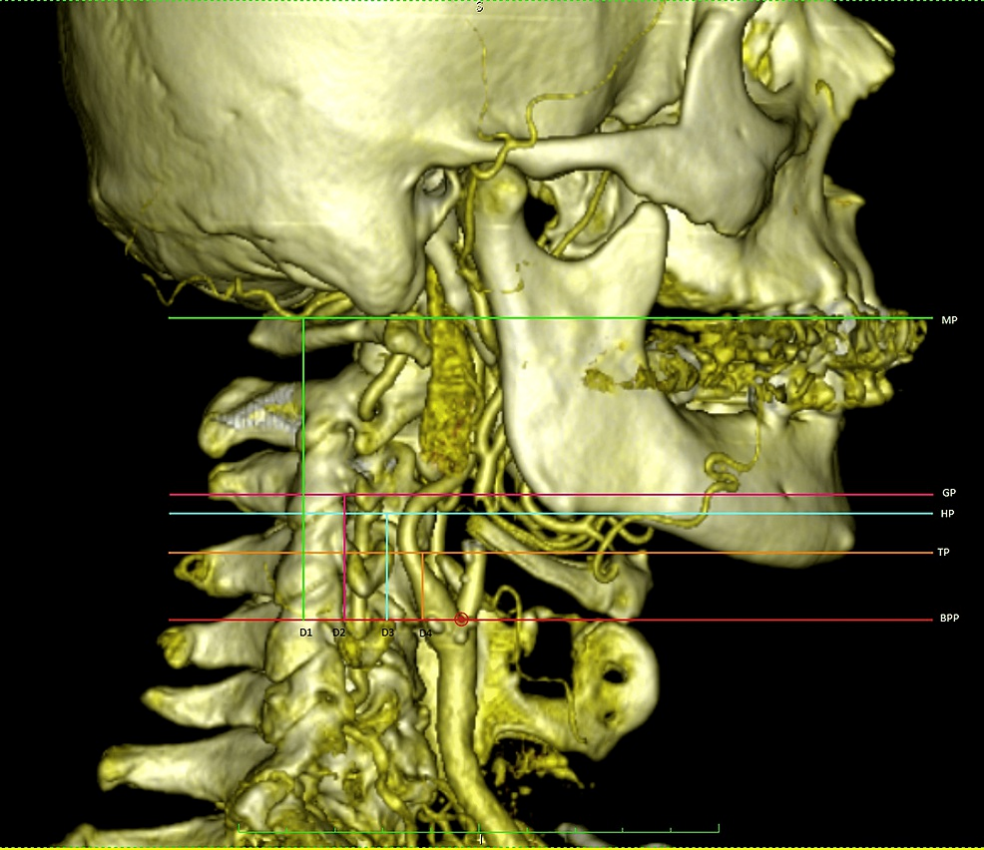
Evaluation of the distance and position of the carotid bifurcation level relative to the surrounding anatomical structures. MP: mastoid plane passing through the lower border of mastoid process, GP: gonional plane passing through the goinon point, HP: hyoid plane passing through the superior border of the greater horn of the hyoid bone, TP: thyroid plane passing through the superior border of the superior horn of the thyroid cartilage, BP: bifurcation point, BPP: birfurcation point plane, D1: distance between BPP and MP, D2: distance between BPP and GP, D3: distance between BPP and HP, D4: distance between BPP and TP.

In the 3D reconstructions obtained, the head and neck were viewed fully laterally. The Frankfurt Horizontal Plane was used to bring the images to the natural head position [18]. The point of separation of ICA and ECA was called the bifurcation point. The distance between the transverse plane passing through the BP and the defined planes of the surrounding structures was measured in millimeters (mm) (Figure 1). Relevant structures 2 mm below or above the BP from these planes were considered to be at the same level as the CB. Others were classified as above or below CB based on their position.

Measurements from the anterior side

**Figure 2:**
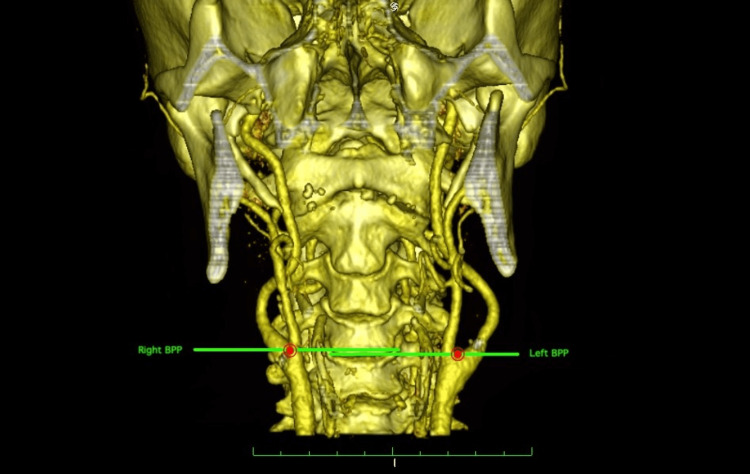
Evaluation of carotid bifurcation level according to cervical vertebrae. BP was determined in the anterior view and the transverse plane passing through it was drawn for both sides. The intersection points of these planes on the cervical vertebrae were evaluated.

The level of the CB relative to the cervical vertebrae was determined in a way similar to a customized leveling method [19]. Vertebral bodies were divided transversely into three equal levels: the upper 1/3, middle 1/3, and lower 1/3. Each intervertebral disc was considered a level (Figure 2).

Statistical analysis

The conformity of the data to the normal distribution was tested with the Shapiro-Wilk test. Mann Whitney U test was used to compare the non-normally distributed features in two groups, and Kruskal-Wallis and Dunn multiple comparison tests were used to compare more than two independent groups. The Spearman correlation coefficient was used to test the relationships between numerical variables, and the chi-square test was used to test the relationships between categorical variables. The SPSS for Windows version 24.0 package program (IBM SPSS, Armonk, NY) was used for statistical analysis, and p<0.05 was considered statistically significant.

## Results

In all cases, the CB was observed below the mastoid process. D1 did not show a significant difference between the genders on the left side. However, when looking at the right side and right-left together, it was seen that this distance was greater in males than in females. D2 did not show a significant difference between the sexes when viewed on the right, on the left, and on the right-left together. It was observed that the D3 distance was higher in females than in males when viewed on the right, on the left, and on the right-left together. On average, CB was observed above the thyroid cartilage in males and females. D4 did not show a significant difference between the genders on the right side. However, when looking at the left side and right-left together, it was seen that this distance was greater in males than in females (Table 2).

**Table 2:** Distribution of the mean and standard deviation (S.D.) values of the distance parameters measured laterally by gender. *Significant at 0.05 level, D1: distance between CB and mastoid process (mm), D2: distance between CB and Gonion (mm), D3: distance between CB and hyoid bone (mm), D4: distance between CB and thyroid cartilage (mm), negative values indicate that CB is below the relevant plane, positive values indicate that CB is above the relevant plane.

Parameter (mm)	Mean ± SD		p-value	Mean ± SD
	Female (n=123)	Male (n=124)		Total (n=247)
Right				
D1	−48.59±9.55	−52.33±10.93	0.005*	−50.47±10.41
D2	−17.29±9.1	−19.3±10.79	0.115	−18.3±10.01
D3	−6.37±9.17	−3.8±10.81	0.045*	−5.08±10.09
D4	4.6±11.46	7.48±12.2	0.057	6.05±11.9
Left				
D1	−49.14±10.96	−51.68±11.22	0.073	−50.42±11.14
D2	−17.6±10.86	−19.14±11.41	0.279	−18.38±11.14
D3	−6.47±10.69	−3.13±10.52	0.014*	−4.79±10.71
D4	4.44±11.95	8.66±12.66	0.008*	6.56±12.46
Parameter (mm)	Female (n=246)	Male (n=248)	p−value	Total (n=494)
Right-left together				
D1	−48.87±10.26	−52±11.06	0.001*	−50.44±10.77
D2	−17.66±9.77	−19.01±11.31	0.159	−18.34±10.58
D3	−6.42±9.94	−3.47±10.65	0.002*	−4.94±10.4
D4	4.52±11.68	8.07±12.42	0.001*	6.3±12.17

When looking at the right side and right-left together, being below the os hyoideum was highly significant in females, while being above it was highly significant in males. There was no significant relationship between other position parameters and genders (Figure 1 and Table 3).

**Table 3:** Position of carotid bifurcation relative to anatomical structures. *Significant at 0.05 level, P1: CB's position relative to mastoid process, P2: CB's position relative to Gonion, P3: CB's position relative to hyoid bone, P4: CB's position relative to thyroid cartilage.

Parameter			Mean ± SD (mm)	Total		Gender				p-value
						Female		Male		
				n	%	n	%	n	%	
P1	Right (n=247)	Below	50.47±10.41	247	100	123	100	124	100	1
		Same level	-	-	-	-	-	-	-	
		Above	-	-	-	-	-	-	-	
	Left (n=247)	Below	50.42±11.14	247	100	123	100	124	100	1
		Same level	-	-	-	-	-	-	-	
		Above	-	-	-	-	-	-	-	
	Right-left together (n=494)	Below	50.44±10.77	494	100	246	100	248	100	1
		Same level	-	-	-	-	-	-	-	
		Above	-	-	-	-	-	-	-	
P2	Right (n=247)	Below	19.13±9.21	238	96.4	120	97.6	118	95.2	0.545
		Same level	0.98±1.38	5	2	2	1.6	3	2.4	
		Above	6.73±3.68	4	1.6	1	0.8	3	2.4	
	Left (n=247)	Below	19.21±10.41	238	96.4	121	98.4	117	94.4	0.239
		Same level	0.66±1.3	4	1.6	1	0.8	3	2.4	
		Above	7.36±5.89	5	2	1	0.8	4	3.2	
	Right-left together (n=494)	Below	18.66±10.18	476	96.4	239	97.2	237	95.6	0.236
		Same level	0.25±1.53	9	1.8	5	2	4	1.6	
		Above	7.08±4.74	9	1.8	2	0.8	7	2.8	
P3	Right (n=247)	Below	10.93±6.47	161	65.2	90	73.2	71	57.3	0.018*
		Same level	0.18±1.58	23	9.3	11	8.9	12	9.7	
		Above	7.93±4.87	63	25.5	22	17.9	41	33.1	
	Left (n=247)	Below	11.32±7.67	151	61.1	84	68.3	67	54	0.07
		Same level	0.24±1.47	28	11.3	11	8.9	17	13.7	
		Above	7.63±5.05	68	27.5	28	22.8	40	32.3	
	Right-left together (n=494)	Below	11.12±7.07	312	63.2	174	70.7	138	55.6	0.002*
		Same level	0.21±1.5	51	10.3	22	8.9	29	11.7	
		Above	7.78±4.95	131	26.5	50	20.3	81	32.7	
P4	Right (n=247)	Below	8.14±5.5	67	27.1	38	30.9	29	23.4	0.385
		Same level	0.22±1.6	23	9.3	10	8.1	13	10.5	
		Above	12.96±8.39	157	63.6	75	61	82	66.1	
	Left (n=247)	Below	9.57±6.67	62	25.1	36	29.3	26	21	0.1
		Same level	0.44±1.54	17	6.9	11	8.9	6	4.8	
		Above	13.13±8.27	168	68	76	61.8	92	74.2	
	Right-left together (n=494)	Below	8.83±6.11	129	26.1	74	30.1	55	22.2	0.104
		Same level	0.31±1.56	40	8.1	21	8.5	19	7.7	
		Above	13.05±8.32	325	65.8	151	61.4	174	70.2	

**Figure 3:**
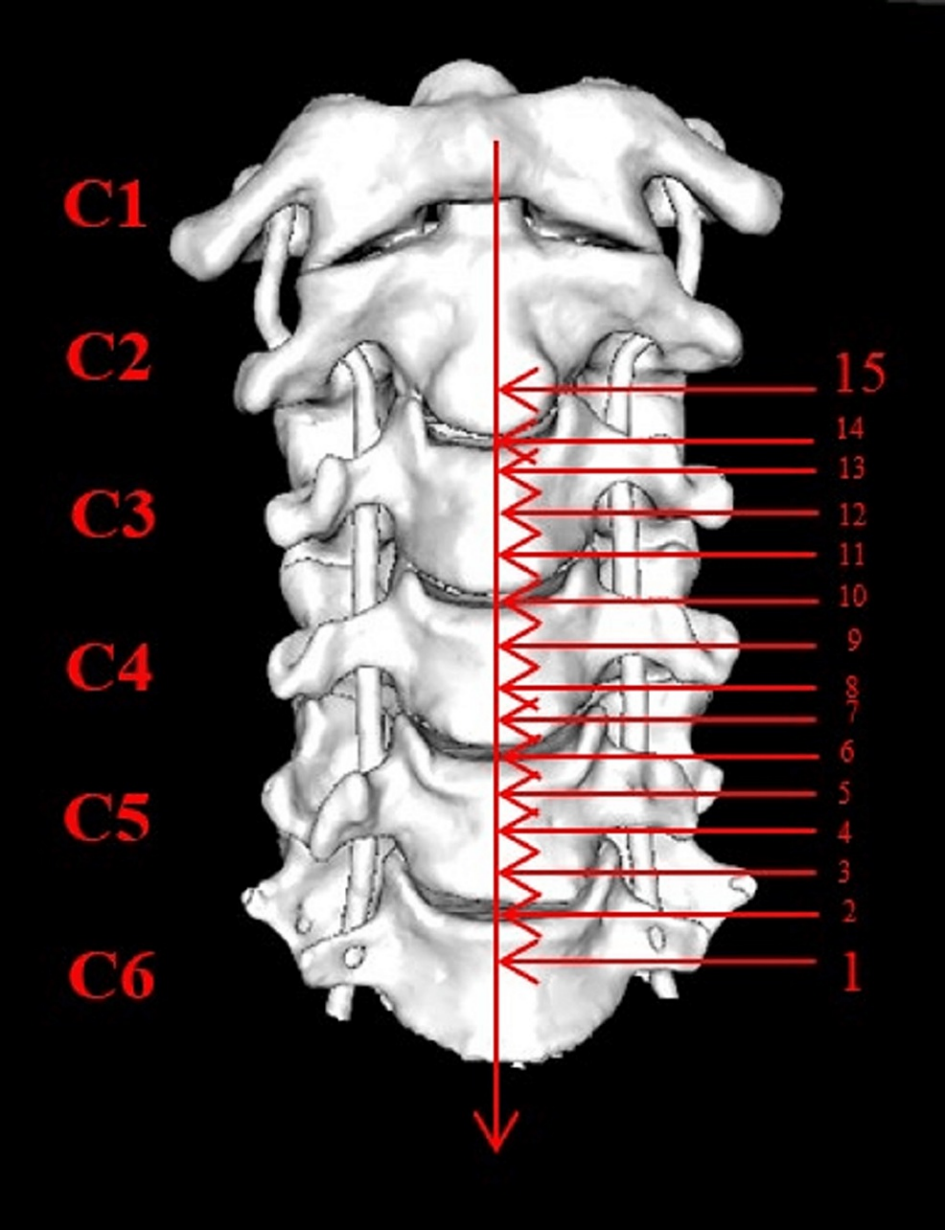
Lowest (1) and highest (15) vertebral levels at which carotid bifurcation is observed.

**Figure 4:**
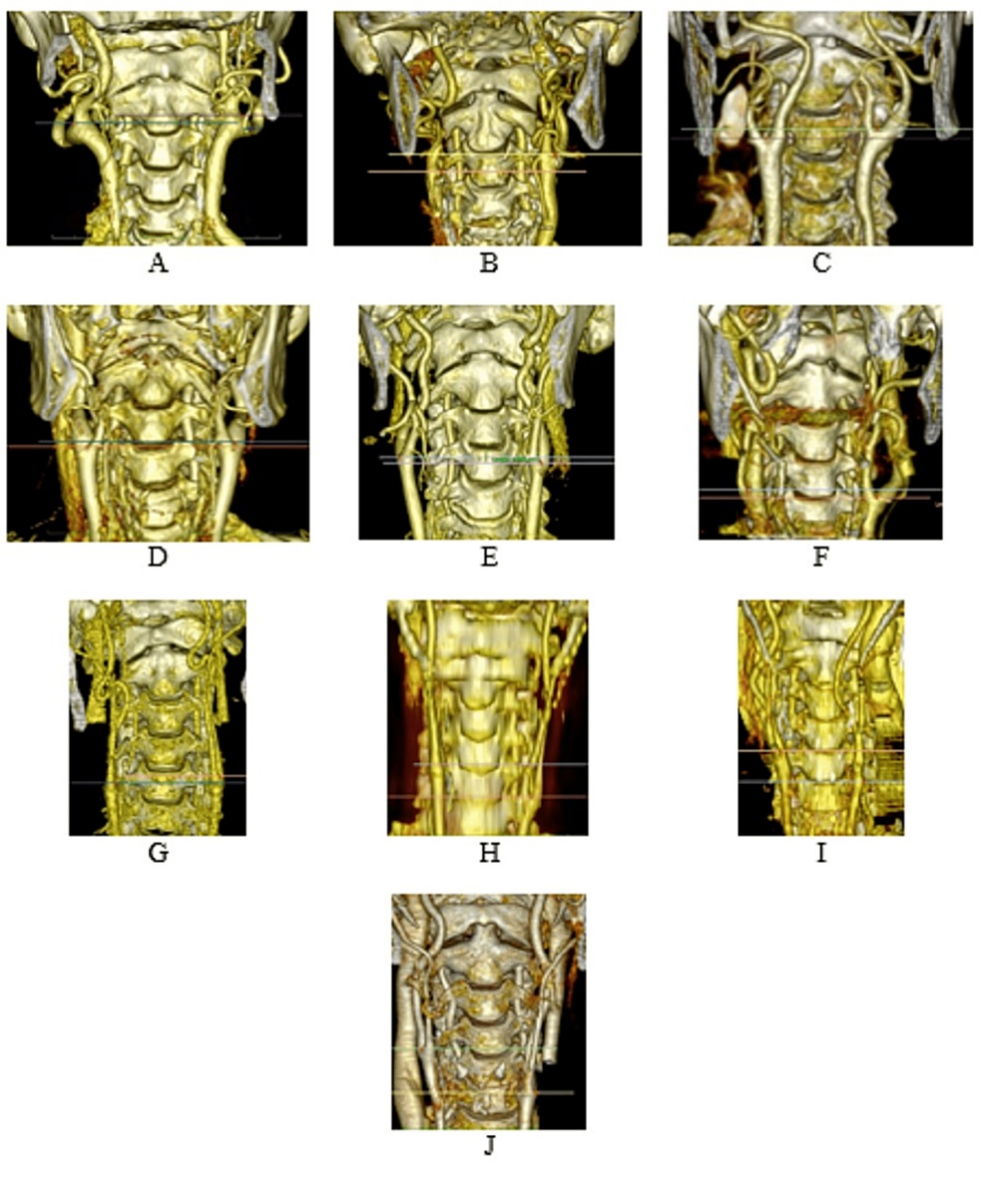
Various vertebral levels where carotid bifurcation is observed. (A) CB bilaterally at C2 lower level (15), (B) CB at right C3 middle, left at C2-3 discus level, (C) CB at right C3 middle (12), left C3 upper (13) level, (D) CB right C3- 4 discus (10), left C3 lower (11) level, (E) CB right C4 upper (9), left C4 middle (8) level, (F) CB right C4 lower (7), left C4-5 discus (6) level, (G) CB right C5 middle (4), left C5 upper (5) level, (H) CB right C4-5 discus (6), left C5 lower (3) level, (I) CB right C4-5 discus (6), at the level of C5-6 discus (2) on the left, (J) CB at the level of C4 lower (7) on the right, C6 upper (1) on the left. As seen in the examples, the CB level in humans was generally (76.1%) unsymmetrical.

Carotid bifurcation was seen at 15 levels in total, the lowest in the upper 1/3 of the C6 vertebral body (level 1, C6 upper), and the highest in the lower 1/3 of the C2 vertebral body (level 15, C2 lower) (Figures 3-4 and Table 4).

**Table 4:** Distribution of the level of the CB according to the vertebrae cervicales by gender. *Significant at the 0.05 level, F: female (n=123), M: male (n=124), T: total (n=247).

		Right				Left				Right-left together			
Verterbal level	No	F	M	p	T	F	M	p	T	F	M	p	T
C2 vertebral body’s lower 1/3	15	3	7	0.362	10	5	6	0.607	11	8	13	0.382	21
C2-3 intervertebral disc	14	2	3		5	4	6		10	6	9		15
C3 vertebral body’s upper 1/3	13	14	7		21	11	10		21	25	17		42
C3 vertebral body’s middle 1/3	12	18	13		31	13	14		27	31	27		58
C3 vertebral body’s lower 1/3	11	17	18		35	25	20		45	42	38		80
C3-4 intervertebral disc	10	24	18		42	16	17		33	40	35		75
C4 vertebral body’s upper 1/3	9	14	21		35	20	12		32	34	33		67
C4 vertebral body’s middle 1/3	8	8	13		21	7	17		24	15	30		45
C4 vertebral body’s lower 1/3	7	7	11		18	9	10		19	16	21		37
C4-5 intervertebral disc	6	10	11		21	6	7		13	16	18		34
C5 vertebral body’s upper 1/3	5	5	1		6	2	3		5	7	4		11
C5 vertebral body’s middle 1/3	4	1	1		2	1	2		3	2	3		5
C5 vertebral body’s lower 1/3	3	-	-		-	1	-		1	1	-		1
C5-6 intervertebral disc	2	-	-		-	1	-		1	1	-		1
C6 vertebral body’s upper 1/3	1	-	-		-	2	-		2	2	-		2

Carotid bifurcation was not observed at the C6 upper, C5-6 discus, and C5 lower levels on the right and left sides in males; it was not observed only on the right side in females. The most common level on the right side was C3-4 discus in females, C4 upper in males, and C3-4 discus in total. When looking at the left and right-left sides together, the most common level was the C3 lower level. Vertebral levels did not differ significantly between genders (Table 4).

There was a weak positive correlation between D1 and age only in females when viewed on the right, left, and right-left together. The correlation between D3 and age was the same as for D1. There was a weak positive correlation between D2 and age only on the right side and only in females. There was a weak negative correlation between D4 and age when looking at the right side, left side, and right-left together, only in males. While there was a positive but very weak correlation between the right-side vertebral level and age, there was no correlation between the left-side vertebral level and age (Table 5).

**Table 5:** Distribution of correlations between distance parameters and age by gender. *Significant at 0.05 level, **significant at 0.01 level, D1: distance between CB and mastoid process, D2: distance between CB and Gonion, D3: distance between CB and hyoid bone, D4: distance between CB and thyroid cartilage.

Parameter	Gender	Right		Left		Right-left together	
		p-value	r	p-value	r	p-value	r
D1	Female	0.005	0.249**	0.023	0.205*	0.001	0.225**
	Male	0.422	-	0.665	-	0.382	-
	Total	0.040	0.131*	0.093	-	0.008	0.118**
D2	Female	0.038	0.187*	0.246	-	0.382	-
	Male	0.486	-	0.706	-	0.135	-
	Total	0.095	-	0.343	-	0.065	-
D3	Female	0.007	0.244**	0.038	0.187*	0.001	0.213**
	Male	0.323	-	0.735	-	0.343	-
	Total	0.006	0.174**	0.045	0.128*	0.001	0.150**
D4	Female	0.770	-	0.401	-	0.417	-
	Male	0.033	-0.192*	0.026	−0.200*	0.002	-0.196**
	Total	0.141	-	0.071	-	0.020	-0.105*
Vertebral level	Female	0.027	0.199*	0.148	-	0.009	0.165*
	Male	0.143	-	0.372	-	0.098	-
	Total	0.014	0.156*	0.110	-	0.004	0.128*

There was a strong positive correlation between the right and left D1, D2, D3, and D4 values. There was a similar correlation between the right and left vertebral levels (Table 6).

**Table 6:** Distribution of correlations between right and left side distance parameters by gender. *Significant at 0.05 level, **significant at 0.01 level, D1: distance between CB and mastoid process, D2: distance between CB and Gonion, D3: distance between CB and hyoid bone, D4: distance between CB and thyroid cartilage.

Parameter	Gender	p-value	r
Right-left D1	Female	0.001	0.776**
	Male	0.001	0.691**
	Total	0.001	0.733**
Right-left D2	Female	0.001	0.728**
	Male	0.001	0.739**
	Total	0.001	0.734**
Right-left D3	Female	0.001	0.741**
	Male	0.001	0.753**
	Total	0.001	0.749**
Right-left D4	Female	0.001	0.802**
	Male	0.001	0.786**
	Total	0.001	0.797**
Right-left vertebral level	Female	0.001	0.761**
	Male	0.001	0.786**
	Total	0.001	0.773**

When the symmetry of CB was examined, it was observed that 59 out of 247 individuals were located symmetrically. The right CB level was higher in 83 individuals and the left CB level was higher in 105 individuals (Table 7 and Figure 4).

**Table 7:** Symmetry of the vertebral level of the carotid bifurcation.

	Female (n=123)		Male (n=124)		Total (n=247)	
	n	%	n	%	n	%
Higher right CB	47	38.2%	36	29.0%	83	33.6%
Higher left CB	51	41.5%	54	43.5%	105	42.5%
CB levels are symmetrical	25	20.3%	34	27.4%	59	23.9%

## Discussion

According to the literature review we have done, there are many studies in which CCA does not form a bifurcation and gives ECA branches directly [1,20-22]. In this study, all CCAs split into terminal branches, forming CB.

Superficial anatomical signs are very important for clinical examination, radiological imaging, diagnosis, treatment planning, surgical interventions, and understanding of anatomy [16,17]. It is an important criterion to externally determine the CB level during the clinical examination of the neck [1]. Accurate determination of the CB level can also be useful in planning surgical interventions and estimating technical difficulties. The great arteries of the neck region have many variabilities, and the confusion about these arteries and the lack of knowledge and experience on this subject can lead to fatal results [9,23]. The level of CB has been described in the classical literature as C3-4 intervertebral disc or C4 vertebral body, which corresponds to the upper border of the thyroid cartilage [16].

Measurements from the lateral side

There were studies evaluating the level of CB according to the mastoid process, gonion, hyoid bone, and thyroid cartilage [1,4,5,9,16,17,24-27]. Some of them evaluated this relationship as a relative position [5,9,17,24], some by specifying distance [25,27], and some as a combined [1,4,16,26]. Two of these studies were CTA [4, 16], and the others were cadaveric studies.

McNamara et al. [4] evaluated 140 carotid bifurcations in three dimensions in their study on CTA images of 76 individuals. Our literature review was the only study that measured the distance of the CB to the mastoid process. As expected, all CBs were located below the mastoid process and were highly correlated with the high bifurcation. We can state that the measured distance values are compatible with the measured distance values in this study.

Some researchers [1,4,26,27] have measured the distance between CB and Gonion point. Among the studies mentioned, the research that is compatible with this study is the work of Ribeiro et al. [27]. The values found by McNamara et al. [4] are close to the values of this study. However, the values of Klosek et al. [1] and Özgür et al. [26] are almost double the values of this work. Among the studies measuring the distance between CB and Gonion, two of them measured these values separately for the right and left sides [1,26]. They found no significant difference between the right and left sides in either study. In the study of Klosek and Rungruang [1], which is the only study comparing this distance between the sexes, they stated that the distance between the CB and Gonion is greater on the left side in males than in females. Similarly, in this study, no significant difference was found between the right and left sides or between the genders for this value. They noted the position of CB relative to Gonion and that 95.7% of the findings by McNamara et al. and 100% of the findings by Klosek et al. were below the plane passing through Gonion. In this study, this rate was determined to be 96.4%, and the results were similar to those of the researchers.

The first of two studies measuring the distance between the CB and hyoid bone was the study by Rafiah et al. [25]. They described this distance as CB was 11-13 mm below and 3-18 mm above the hyoid bone. We can say that the specified values are compatible with this study. In another study, Özgür et al. [26] found no significant difference between them, similar to this study, when comparing this distance value as right and left. The distance values ​​measured by Özgür et al. were greater than the findings of this study. In the current study, unlike the literature, the distance between the CB and the hyoid bone was also compared between the sexes and found to be higher in females than in males. We obtained parallel results with one of the three studies evaluating the position of the CB relative to the hyoid bone [4]. In one study, it was stated that 87.5% [9] of CBs were located below the hyoid bone, a higher rate than our study. In a case study, it was reported that CB was bilaterally at the level of the hyoid bone [17].

Three of the four studies measuring the distance between the CB and the thyroid cartilage found values greater than the values measured in this study [16,26,27]. One detected rather small values ​​from this study [1]. The fact that the results of the four studies mentioned differ considerably, both among themselves and in this study, and that the reliability of the thyroid cartilage, which is considered a topographic landmark and is widely used to describe the CB level in the sources, has become questionable. In this study, we can suggest that the Gonion, which has a relatively small interindividual standard deviation value, should be used instead of the thyroid cartilage in determining the position of the CB. Mirjalili et al. [16] stated that the CB-thyroid cartilage distance did not show any difference between the sides, but it was smaller in females than in males on the right side. Similarly, there was no difference between the parties in this study. On the contrary, on the left side, these values ​​were smaller in females than in males when we compared them by gender. In the studies of Klosek et al. [1] and Özgür et al. [26], there was no significant difference between the right and left sides in parallel with our study. The difference between our study and that of Klosek et al. was that they did not identify a difference between the sexes. The position of the CB relative to the thyroid cartilage was evaluated as "above" by 70% or more in some studies [1,4,16,24,26]. In one study, this rate was at 55%, while it was reported to be at the same level as 39% [5]. In another study, it was stated that CB was at the same level as the thyroid cartilage, with the highest rate of 50% [9]. In this study, CB was located below the thyroid cartilage, which is consistent with the general literature.

Measurements from the anterior side

The level of CB relative to the cervical vertebrae has been investigated in many studies [1,4,13,19,23,27-35]. In some of these studies, the vertebral body was accepted as three levels, as in our study: the lower 1/3, the middle 1/3, and the upper 1/3 [4,13,19]. In one study, the vertebral body was accepted as having two levels, the lower 1/2 and the upper 1/2 [28]. In other studies, the vertebral body was accepted as a single level. In the literature, according to the cervical vertebrae, the CB level can vary between the middle 1/3 of the C2 vertebral body and the level of the C7 vertebral body.

In parallel with the study by McNamara et al. [4], this study found the highest and lowest levels of CB as C2 lower 1/3 and C6 upper 1/3, respectively. De Syo et al. [19] reported the lowest and highest levels as C3 upper 1/3 and C6 lower 1/3, respectively. Both studies reported the most common vertebral CB level as C4 middle 1/3. Smith and Larsen [28] determined the lowest and highest vertebral levels as C2 lower 1/2 and C5 lower 1/2 and reported the most common vertebral level as C3-4 disc. Hayashi et al. [13] reported the lowest and highest vertebral levels as C2 middle 1/3 and C4-5 disc, and the most common vertebral level as C3 middle 1/3 on both the right and left sides. They found no significant difference between the right and left vertebral levels. However, as an important finding, they reported a 3.99-mm level difference between the angiographic level of CB and the level observed during surgery. In other words, the CB level observed during the surgical intervention will be slightly lower than the level determined in the preoperative angiograms.

Among the studies accepting the vertebral body as a single level, Anangwe et al. [29], Anu et al. [30], and Ribeiro et al. [27] reported the highest vertebral level of the CB as the C2 body, while the lowest level was found as the C6-7 disc, the C5 body, and the C4 body, respectively. Anangwe et al. and Anu et al. reported the most common level as C3 body, while Ribeiro et al. reported that the C2 body level was found to be higher. Klosek et al. [1] and Woldeyes [31] determined the lowest vertebral levels as C5 body and C4 body, respectively. Civelek et al. [32] and Espalieu et al. [33] found this level to be the C4-5 disc. They found the highest vertebral level of CB as a C2-3 disc. The most common level of CB is reported by Woldeyes as a C3 body; Civelek and Espalieu reported it as a C4 body. Klosek et al. reported that the level of CB in females was at the level of the C4 body on the right and the C3-4 disc on the left. They also reported that the CB was at the level of a C4-5 disc on the right side and a C4 body on the left side in males. While Zümre et al. [23] reported the CB level in a rather narrow segment, between the C3 body and the C5 body, they reported the most common level of CB as the C3 body level on both the right and left sides. Ito [35] described the CB level as being at the level of the C4 body, above the C4 body, and below the C4 body. These researchers reported the most common level of CB in the C4 body in males and females.

The most common level of CB in this study was the C3 lower 1/3 level. The CB level did not differ significantly between the genders or between the right and left sides. When the CB level was evaluated together with the right and left, it increased in direct proportion to the age in females, but this was not the case in males. The works of McNamara et al. [4], De Syo et al. [19], and Hayashi et al. [13] were similar to this study in the method of measuring vertebral levels of CB. The most common vertebral levels of CB found in this study were consistent with the study of Hayashi et al. As in this study, high CB levels were more common than low CB levels in all studies.

In the studies in the literature, the symmetry of CB was examined, and quite different results were obtained from each other and from this study (Table 8).

Limitations

There were some limitations in this study. As far as possible, patients without stenosis and without surgical and/or interventional intervention were included. However, our study may not be as representative of the normal population as it is generally performed on sick individuals. On the other hand, it would be unethical to expose healthy individuals to unnecessary radiation. Although this study was conducted in a large group, it should be followed in larger populations in order to support the findings obtained. The study is limited to the borders of the Republic of Turkey. New studies should be conducted to evaluate the similarities or differences between different races.

## Conclusions

All these values, which emerged as a result of the study, provide general information about the topography of the CB according to the neighboring structures. When we examined the right side, left side, and right-left side together and looked at both genders, it can be said that the standard deviation value of the distance between the CB and the distance between the CB and hyoid bone can be measured with less error than the other two. In other words, estimating the location of the CB according to these two structures will give a more accurate result. The vertebral level of CB did not show any difference in terms of gender and right-left sides. In fact, there was a strong positive correlation between the vertebral levels on both sides. While the vertebral level on the right side increased in direct proportion to age, there was no similar relationship on the left side. It is necessary to be aware of these anatomical variations in order to prevent various iatrogenic complications.
